# Draft Genome Sequence of *Lysinibacillus* sp. Strain BPa_S21, Isolated from Petroleum-Contaminated Soil in Delta State, Nigeria

**DOI:** 10.1128/mra.00456-22

**Published:** 2022-07-21

**Authors:** Grace A. Okenmor, Richard J. Kutshik, Ishaya Y. Longdet

**Affiliations:** a Department of Biochemistry, University of Jos, Jos, Nigeria; University of Southern California

## Abstract

*Lysinibacillus* sp. strain BPa_S21 was isolated from petroleum-contaminated soil in Nigeria. Here, we present the draft genome sequence, consisting of a 4,813,249-bp genome with 4,666 protein-coding sequences, 4,580 functionally assigned genes, and 74 RNA genes. An average nucleotide identity-based whole-genome comparison suggests that this is a novel species within the *Lysinibacillus* genus.

## ANNOUNCEMENT

*Lysinibacillus* species are Gram-positive, rod-shaped bacteria from diverse environments (1–3) that exhibit different activities (4, 5). Here, we report the draft genome sequence of *Lysinibacillus* sp. strain BPa_S21 from Nigeria.

The organism was isolated from soil (pH 5.53) collected at a petroleum mining site in Delta State, Nigeria (5.35N, 6.10E). The soil sample was collected to a depth of 5 cm using a soil auger, put into sterile sample bottles, kept in airtight zipped bags, and transported to the laboratory (1). The soil was dried for 5 days, and 1 g was suspended in 9 mL autoclaved distilled water and vigorously shaken to dislodge the microorganisms (1). From this stock solution, serial dilutions (10^−1^ to 10^−10^) were prepared. An aliquot (0.1 mL) from each dilution was plated on a sterile nutrient agar plate and incubated at 37°C for 48 h. Bacterial growth was recorded only from 10^−1^, 10^−2^, and 10^−3^ dilutions. The plate with the 10^−3^ dilution was selected for the study. Based on morphological observations, six colonies were identified, each plated on a nutrient agar plate by streaking, and incubated at 37°C for 24 h. To obtain pure colonies, each colony was streaked on two other nutrient agar plates and incubated at 37°C for 24 h in succession. The isolates were grown overnight in nutrient broth at 37°C. A 2-mL portion was centrifuged at 14,000 × *g* for 3 min, and the supernatant was discarded. This procedure was repeated with a fresh 2-mL portion. To wash the cell pellets, 500 μl of phosphate-buffered saline was added, and the mixture was centrifuged at 14,000 × *g* for 3 min. The washing was repeated.

Genomic DNA was extracted using the ZymoBIOMICS DNA miniprep kit. Default parameters were used for all software unless otherwise specified. DNA libraries were prepared using the Nextera XT DNA library preparation kit (Illumina) and the Nextera index kit (Illumina), and genomic DNA was fragmented using the Illumina Nextera XT fragmentation enzyme. Combinatory dual indexes were added to each sample, followed by 12 cycles of PCR to construct libraries. DNA libraries were purified using AMPure magnetic beads, eluted in Qiagen EB buffer, quantified using a Qubit 4 fluorometer and Qubit double-stranded DNA (dsDNA) HS assay kit, and sequenced on an Illumina HiSeq X Ten platform, producing 5,523,655 read pairs (2 × 150 bp). Raw single-end reads were trimmed and processed using BBDuk (v37.78) (6) with a read quality trimming parameter of 20.

The trimmed fastq reads (with coverage depth of 13.803 million reads) were assembled using SPAdes (v3.13.0) (7), and the completeness was evaluated using CheckM (v1.1.2) (8). This analysis yielded 36 contigs, with an *N*_50_ value of 327,950 bp (contig size range, 575 to 629,221 bp; mean contig size, 133,701.36 bp), a total assembled size of 4,813,249 bp, genome coverage of 430×, and a GC content of 36.79%. The genome was annotated using PGAP (v6.1) (9), which identified 4,666 protein coding sequences, including 4,580 proteins with functional assignments and 86 pseudogenes, 10 rRNA operons, 59 tRNA genes, and 5 noncoding RNA gene. The whole-genome comparison based on computed average nucleotide identity using MUMmer4 (10) revealed about 85% similarity between *Lysinibacillus* sp. strain BPa_S21 and Lysinibacillus fusiformis RB-21 (GenBank accession number GCF_000724775.3), Lysinibacillus sphaericus DSM 28 (GenBank accession number GCF_002982115.1), and Lysinibacillus varians GY32 (GenBank accession number GCF_000600105.1). This finding suggests that *Lysinibacillus* sp. strain BPa_S21 is a novel species within the *Lysinibacillus* genus.

### Data availability.

This genome was deposited in DDBJ/EMBL/GenBank under accession number JALIRS000000000. The version described in this paper is version JALIRS010000000. The raw reads were deposited in the Sequence Read Archive (SRA) under accession number SRR19091828, with BioProject accession number PRJNA815837 and BioSample accession number SAMN26645039.
